# MicroRNAs: an emerging science in cancer epigenetics

**DOI:** 10.1186/2043-9113-3-6

**Published:** 2013-03-16

**Authors:** Rishabh Kala, Gregory W Peek, Tabitha M Hardy, Trygve O Tollefsbol

**Affiliations:** 1 Department of Biology, University of Alabama Birmingham, 1300 University Boulevard, Birmingham, AL 35294, USA; 2 Center for Aging, University of Alabama Birmingham, 1530 3rd Avenue South, Birmingham, AL 35294, USA; 3 Comprehensive Cancer Center, University of Alabama Birmingham, 1802 6th Avenue South, Birmingham, AL 35294, USA; 4 Nutrition Obesity Research Center, University ofs Alabama Birmingham, 1675 University Boulevard, Birmingham, AL 35294, USA; 5 Comprehensive Diabetes Center, University of Alabama Birmingham, 1825 University Boulevard, Birmingham, AL 35294, USA

**Keywords:** miRNA, Biogenesis, Diet, Cancer epigenetics, Bioinformatics software

## Abstract

MicroRNAs (miRNAs) are remarkable molecules that appear to have a fundamental role in the biology of the cell. They constitute a class of non-protein encoding RNA molecules which have now emerged as key players in regulating the activity of mRNA. miRNAs are small RNAmolecules around 22 nucleotides in length, which affect the activity of specific mRNA, directly degrading it and/or preventing its translation into protein. The science of miRNAs holds them as candidate biomarkers for the early detection and management of cancer. There is also considerable excitement for the use of miRNAs as a novel class of therapeutic targets and as a new class of therapeutic agents for the treatment of cancers. From a clinical perspective, miRNAs can induce a number of effects and may have a diverse application in biomedical research. This review highlights the general mode of action of miRNAs, their biogenesis, the effect of diet on miRNA expression and the impact of miRNAs on cancer epigenetics and drug resistance in various cancers. Further we also provide emphasis on bioinformatics software which can be used to determine potential targets of miRNAs.

## Introduction

MicroRNAs (miRNAs) are a group of endogenous small and noncoding RNAs that are approximately 18–25 nucleotides in length that play a critical role in the regulation of gene expression. In the past decade, the biological functions and biogenesis of miRNAs have become popular topics for biomedical research. As expected, miRNA expression is highly correlated with human diseases, such as cancer and other aging associated diseases. miRNAs may function not only as oncogenes but also as tumor suppressors, further implicating their roles as therapeutic targets. Moreover, miRNAs can be used as biomarker or prognostic signature molecules for determining the likely outcome of certain diseases such as cancer. The importance of these small non-coding RNA molecules in predicting the outcome of various cancers has been highlighted. Previous study on human patients emphasizes the substantial role of this relatively newly identified class of RNA molecules as diagnostic and prognostic biomarkers in cancers [1].

## miRNA biogenesis and its mode of action

**Figure 1:**
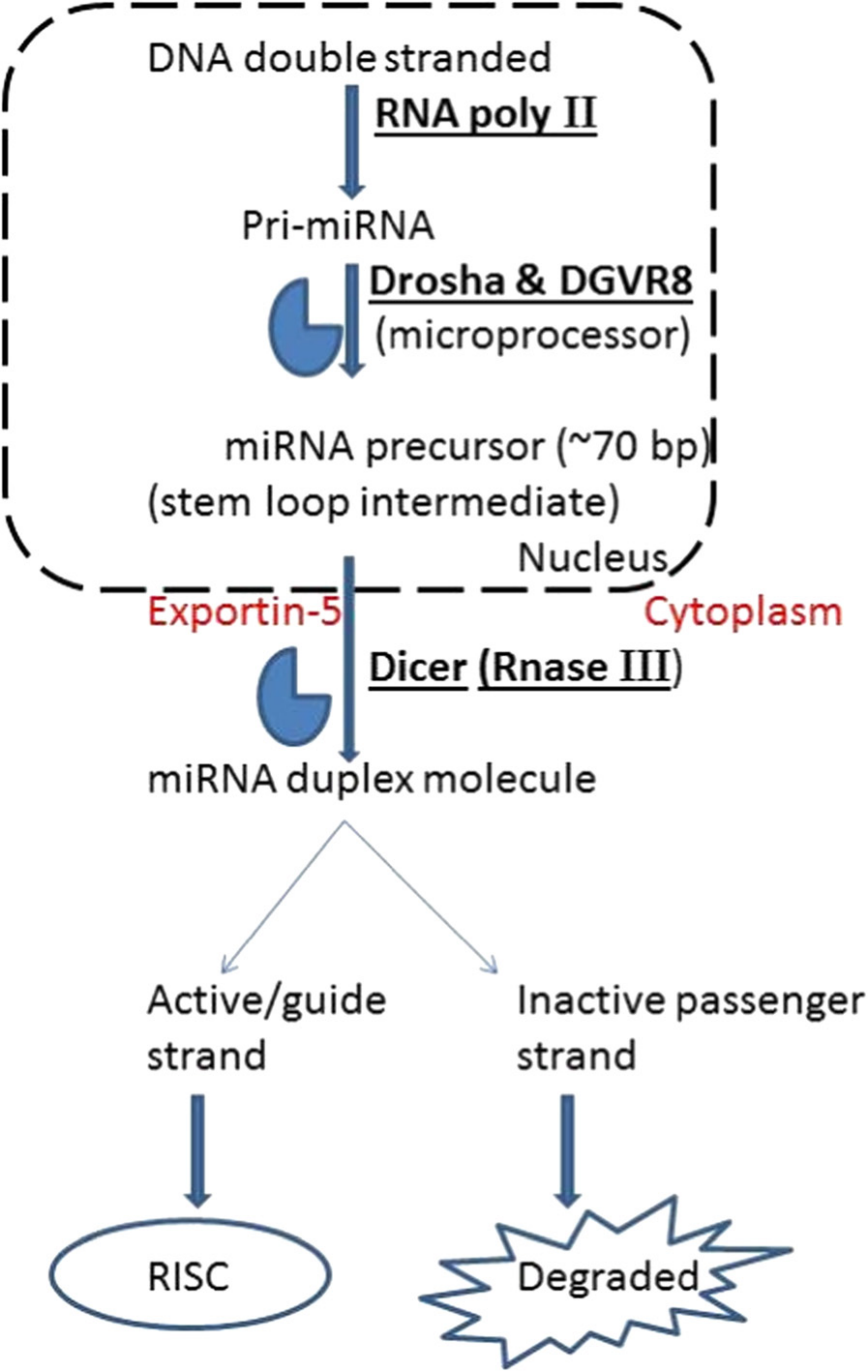
**miRNA biogenesis.** miRNAs undergo a relatively complicated biogenesis starting from a precursor molecule called miRNA, which is further processed by enzymes Drosha and Dicer, to produce a mature/active miRNA molecule.

The miRNAs undergo a relatively complicated biogenesis (Figure 1) that starts with their synthesis as long primary transcripts (pri-miRNA) by RNA polymerase II [2]. This long primary transcript is then further cleaved by Drosha, an RNase III nuclear enzyme which liberates a ~ 60-to 70-nucleotide stem loop intermediate known as the miRNA precursor (pre-miRNAs) [3,4]. Because of the self-complementarity within the RNA molecule, this precursor molecule forms a characteristic hairpin double-strand. The pre-miRNAs are transported from the nucleus to the cytoplasm by Exportin-5, and are further processed by Dicer, a second RNase III enzyme [5]. The role of Dicer is to cleave pre-miRNA molecules to produce 22 basepair dsRNA molecules. One strand (the active, or “guide” strand) is then loaded into the RNA-induced silencing complex (RISC), while the inactive strand, also called a “passenger” strand, is removed and degraded. Through sequence-specific interactions between the mature miRNAs and mRNA, the ribonucleoprotein complex is positioned on either of the two untranslated regions (UTR) of their targets [5-8].

RISC is composed of the transactivation-responsive RNA binding protein (TRBP) and Argonaute (Argo), the catalytic component of RISC complex. The guide strand can then recognize the complementary sequence of mRNA via its association with RISC. miRNA target recognition involves base-pairing between nucleotides 2–7 (the seed) of the 5^′^ end of the miRNAs and a 6, 7 or 8 nucleotide sequence of the mRNA 3^′^ UTR, with additional less absolute requirements [9,10]. These include (1) the fidelity of seed base-pairing to short 3^′^ UTR motifs, (2) high A and U content in the nucleotides surrounding the seed-binding motif, (3) location of the 3^′^ UTR binding site at least 15 nucleotides past the stop codon, (4) seed-binding motif avoidance of any location near the center of a long 3^′^ UTR, and (5) seed-binding motif location close to supplemental UTR pairing with miRNA nucleotides 13–16 [9]. Although it is known that a single species of miRNA can affect the expression levels of many genes, it is not yet clear how the specificity of this function of miRNAs is regulated. These non-coding RNA molecules are evolutionarily conserved and can be located in the introns or exons of genes, or in the sequence between genes (intergenic sequence) and are assumed to be involved in development, cell differentiation, metabolic pathways, signal transduction, proliferation, and apoptosis [6].

During the process of biogenesis there are a host of mechanisms that govern the transcription and post-transcriptional regulation of the miRNAs. The discovery of these mechanisms has enhanced our understanding of miRNA deregulation in a variety of disease states, including cancer, although much work remains for a more complete understanding of miRNA-mediated regulation of gene expression and downstream effects. Examples involve regulatory proteins Dicer and Drosha, with down-regulation of Dicer and Drosha believed to affect miRNA expression level and increase risk in neuroblastoma tumors. In fact, an important study has shown that *in vitro* knockdown of Dicer and Drosha promoted the growth of neuroblastoma cell lines [11].

miRNAs have emerged as new targets in biomedical studies because of their effects on a number of biological phenomena with reported impact on various diseases including ageassociated diseases such as cancer. In light of miRNA involvement in cancer-associated genomic alterations, high-throughput technologies for assessing miRNAs have been developed to study the global miRNA expression patterns in cancer called the miRNAome (Table 1). With the onset of next-generation sequencing, the repertoire of experimentally verified mature miRNA has rapidly expanded [12,13]. Current methodology, as well as an extensive miRNA database, is presented or identified by miRBase, available at http://microrna.sanger.ac.uk/ or http://www.mirbase.org/. It is maintained by the University of Manchester and can be searched by accession number, name, keyword, chromosome location, tissue expression, sequence, homologous sequence or PubMed ID. miRBase is updated frequently with the recently described version miRBase 16 containing over 17,000 miRNA sequences from over 140 species updated in August 2012 (miRBase 19) to over 25,000 mature sequences [13].

**Table 1:** Databases and software used in miRNA analysis

**Database or software**	**Principal applications**	**Additional features**	**Search by**
**miRBase** (http://microrna.sanger.ac.uk/ or http://www.mirbase.org/)	miRNA target identification	Links to databases and software, such as miRanda	Accession number, name, keyword tissue, sequence PubMed ID
**miRanda** (http://microrna.org)	Identification of target genes or targeting miRNA. miRNA expression profile and distinguish conserved and non-canonical sites	Support vector regression (SVR) algorithm to determine level of gene down-regulation.	miRNA, target gene
**TarBase** (http://microrna.gr/)	miRNA target identification		miRNA
**miR2Disease** (http://www.miR2Disease.org)	Associate human diseases with specific miRNA dysregulation	Uses TarBase	Specific miRNA, disease name, tissue or target gene
**TransmiR** (http://cmbi.bjmu.edu.cn/transmir)	Identify transcription factors that regulate specific miRNA	Links to NCBI gene and protein data and literature	Transcription factor names, miRNA name, species, regulation type, literature
**miRTarBase** (http://miRTarBase.mbc.nctu.edu.tw/)	Document experimentally confirmed miRNA-target interactions	Provides method of interaction confirmation, information on mature miRNA and precursor, expression profile, gene target network	Species, miRNA, target gene combination

Useful software for determining miRNA targets has now begun to proliferate [14-16]. Software of particular interest is miRanda, available at http://microrna.org or via miRBase (miRBase Targets) [15,17,18]. miRanda uses data from the genomic database Ensembl, available at http://www.ensembl.org/[17,19], which allows a user to determine the target genes of a specific miRNA, the miRNAs for which a specific gene is a target and the expression profile of a specific miRNA [15]. miRanda accommodates the identification of both conserved and nonconserved target sites which can be individually evaluated by the support vector regression (SVR) algorithm for degree and rank of capacity for gene down-regulation [20]. The mirSVR algorithm was designed for ranking the level of down-regulation associated with miRandadesignated target sites according to miRNA transfection and inhibition experiments [9,20]. The mirSVR scores, which simulate down-regulation predictions, were found to be especially valuable for recognizing gene down-regulation by multiple miRNAs, and they are provided on the miRanda website [20].

The database miR2Disease provides extensive inventory and documentation of involvement of miRNA dysregulation in human disease and is available at http://www.miR2Disease.org[21]. As of the March 2011 update, miR2Disease provided comprehensive documentation of miRNA dysregulation of 349 miRNAs associated with 163 diseases including age-associated disease like cancer [21]. Access is provided by search via specific miRNA, disease name (and associated tissue) or experimentally validated target gene (from TarBase). Detailed analysis is provided by links to referenced literature.

The database TransmiR, available at http://cmbi.bjmu.edu.cn/transmir, documents miRNA regulation by transcription factors and thus provides a critical link to origins of gene dysregulation by miRNA and any consequent disease etiology [22]. As of the March 2012 update, TransmiR documents 201 transcription factors and 209 miRNAs from 16 organisms [22]. It can be assessed by combinations of transcription factor name, miRNA name, species, regulation type (activate or repress) and/or PubMedID. Links are provided to NCBI gene and protein data along with associated literature and networks of transcription factors and their target miRNA genes are included [22].

miRTarBase at http://miRTarBase.mbc.nctu.edu.tw/ is an extensive database of experimentally confirmed miRNA-target interactions. These interactions are experimentally validated using Western blot, knockdown or reporter gene analysis [23]. The miRTarBase database released in October 2011 included 669 miRNAs and 2553 target genes of 14 species [23]. The networks of mature miRNA and gene targets appear to be suitable for potential integration with protein-protein interaction networks as well as network data derived from TransmiR [22,23].

## miRNAs and cancer epigenetics

miRNAs are emerging as a new class of molecules whose deregulation may ultimately contribute to cancer formation. They also likely cooperate with the other classic oncogenes and/or down-regulate tumor suppressor genes in cancer cells to drive the behavior of the tumors. Although many miRNAs have been shown to be deregulated in cancers, the set of miRNAs that actually play a pathogenic role in cancer has not yet been clearly determined. Additional changes in the expression level of miRNAs in cancer cell lines can directly regulate certain fundamental behaviors of cancer cells, such as proliferation and apoptosis [24]. Many of the miRNAs deregulated in cancers have been shown to have a direct impact on tumor suppression and their metastasis. These non-coding classes of RNA can serve as useful biomarkers and may greatly improve clinical management by better defining appropriate treatment options for patients [5].

In addition to their role in tumor suppression or tumor promotion, miRNAs have also been identified as master regulators of key genes implicated in mechanisms of chemoresistance. There are two main mechanisms which are thought to be the key players in chemoresistance: one is genetic and the other is epigenetic. Although evidence regarding genetic changes following chemotherapeutic treatment is limited, numerous studies have demonstrated significant epigenetic alterations in drug-resistant cancer cells [25,26]. In addition to these well-studied mechanisms of cancer drug resistance, there have been recent studies that link cancer drug resistance with the alteration of miRNAs expression [27].

Epigenetics is the study of heritable changes in gene expression caused by mechanisms other than changes in the underlying DNA sequences, which might affect various cellular phenomena like cell signaling, proliferation, apoptosis. Epigenetic processes are commonly thought to favor cell survival and tumor progression. Examples of epigenetic changes are DNA methylation and histone modifications, both of which serve to regulate gene expression without altering the underlying DNA sequence [28,29]. In order for DNA to undergo methylation and histone modifications, epigenetic modifying enzymes such as DNA methyltransferases (DNMTs), histone deacetylases (HDACs), histone acetylases (HAT) and histone demethylases (HDMs) are required. Interestingly, these miRNAs can control the expression of various epigenetic-modifying enzymes which are involved in carcinogenic processes [30,31]. There are a number of studies highlighting this connection. One such study was performed by Lujambio et al. in 2008 which showed that hypermethylation of miR-148 resulted in its down-regulation because of positive feedback that exists to reinforce the overexpression of DNMTs in breast cancer cells which resulted in breast tumor growth and metastasis. Furthermore, the reactivation of miR-148 upon treatment with a DNA demethylating agent was associated with reduced tumor growth and inhibition of metastasis [32]. Another study involved comparison between normal lung cell and cancerous cells and reported an expressional difference of miRNA in both the cell types [33]. The *miRNA-29* family (*miRNA-29a,-29b,-29c*), which is down-regulated in cancers, was shown to have some interesting complementarity with the 3′UTR of DNA methyltransferase (DNMT)3A and 3B both of which are known *de novo* methyltransferases. Further investigation determined whether *miR-29s* could target DNMT3A and 3B expression by restoration of *miR-29s.* It was found that the enforced expression of *miR-29s* in lung cancer cell lines restored normal patterns of DNA methylation and induced re-expression of methylation-silenced tumor suppressor genes, thus affecting cancer growth [33].

miRNAs are implicated in several cellular responses to drug exposure, including, but not limited to, drug influx/efflux, cell cycle arrest, DNA repair, and apoptosis, all of which mediate cancer cell survival and tumor progression. There have been a number of miRNAs which are reported to be involved in breast cancer drug resistance, one of which is *miR-101*, which targets EZH2, the enzyme responsible for trimethylating histone H3 lysine 27 to establish a repressive chromatin state. miRNA upregulation has been linked to tamoxifen and fulvestrant resistance [34,35]. Crosstalk may occur between certain classes of miRNAs such as *miR-101*, *miR-206*, and *miR-221/222*, which translationally repress the estrogen receptor alpha (ERα) and could also be responsible for the decreased sensitivity to anti-estrogen drugs [27,34,35]. Further, on comparing ERα-negative breast cancer cells lines such as MDA-MB 468, HS578T and MDAMD-231 with ERα-positive cell lines such as MCF-7, T47D and MDA-MB 361, there was an expressional difference in *miR-221* and *miR-222*. Further analysis revealed that knockdown of these two marker miRNAs partially restored ERα protein expression in ERα protein-negative/ mRNA-positive cells, thus making them a potential biomarker for prognostic as well as therapeutic purposes [27]. These findings indicate a role for miRNAs in regulating estrogen receptor and drug resistance. Furthermore, since a number of miRNAs can target DNA and histone-modifying enzymes, they are likely to affect gene expression on a much broader scope.

The roles of miRNAs in cancers have been extensively investigated in the past few years. Recently, the connection of miRNA and tumor suppressor networks was elucidated. p53, a wellknown tumor suppressor, regulates diverse physical responses to many cancer-related stress signals, which may affect cell proliferation, cell death, DNA repair, and angiogenesis. Thus far results obtained are mixed. In some studies, p53 was found to affect miRNA expression and in other studies miRNAs were found to play a crucial role in p53-mediated tumor suppression [36]. A wide array of miRNAs were found to be affected by the expression level of this key tumor suppressor gene, including in colon cancer were p53 may have a direct role in miRNA expression [37]. In another study, 470 miRNAs were analyzed using microarray and 12 of these were found to be significantly affected by p53 [38]. Moreover, it has been found that *miR-34a* affects the pathway that mediates cellular aging and limits longevity, by mitigating SIRT1 expression and p53-related apoptosis, stability and activity [39]. SIRT1, a mammalian homologue of yeast silent information regulatory Sir2, with an enzymatic activity of nicotinamide adenine dinucleotide (NAD+)-dependent histone deacetylases, is a class III histone deacetylase. *miR-34a* is a tumor suppressor gene that is an evolutionarily conserved miRNA, with a single, recognizable orthologue in several invertebrate species [36]. *miR-34a* functions as a tumor suppressor, in part, through a *SIRT1-p53* pathway. This miRNA inhibits *SIRT1* expression through a miR-34a-binding site within the 3′ UTR of *SIRT1*. In support of this concept, it was discovered that miRNA did not affect the *SIRT1* RNA transcription but it did affect the translation of SIRT1 RNA by acting on the 3′response element of SIRT-1 [39,40]. Xu et al. [40] reported an effort to use softwares such as miRnada, TargetScan, and Pic Tar, which could help in target prediction for *miR-22* and *miR-34a*. Moreover, knockdown of *miR-34a* function by antisense oligonucleotides attenuates the acetylation of p53. *miR-34a* may have other targets besides *SIRT1* that can regulate cell survival. Thus, *SIRT1* may be one of several distinct targets of *miR-34a* that contribute to its ability to promote apoptosis.

Recently, a study was performed with 5-fluorouracil (5-FU)-resistant human colorectal cancer DLD-1 cells and with parental DLD-1 cells [41]. In that study the level of *miR-34a* was observed to be low in the drug resistant cell line but it was found to be high in parental cells after treatment. Moreover with respect to *SIRT-1* expression, *miR-34a* was observed to be upregulated in resistant cells. Further activation of *miR-34a* resulted in inhibition of growth with a decrease in *Sirt1* expression. These findings suggest that *miR-34a* targeting the *Sirt1* genes could negatively regulate, at least in part, the resistance to 5-FU in human colorectal cancer DLD-1 cells [41].

The *miR-200* family is a crucial modulator of epithelial to mesenchymal transition (EMT), which is a normal embryological process involved in various adult pathologies including cancer metastasis and tumorigenicity. The *miR-200* family is down-regulated and exhibits tumor suppressive properties in renal, prostate, breast, bladder, pancreatic, and gastric cancers. It is a key regulator of the epithelial phenotype and is involved in EMT processes in breast cancer. There have been a crosslinking reported between Class III histone deacetylase *SIRT1*, a proposed oncogene in breast cancer, and *miR-200*. With overexpressed *SIRT1* an overexpression of EMT was observed due to a positive feedback loop between epigenetically silenced *miR-200* and *SIRT1*. Further restoration of *miR-200* or the knockdown of *SIRT1* prevented transformation of normal mammary epithelial cells as evidenced by decreased breast cancer metastasis. Finally, it was observed that *SIRT1* overexpression is associated with decreased *miR-200a* in breast cancer patient samples, indicating that *miR-200a* may be a potential tumor suppression target in breast cancer metastasis [42]. Several other class of miRNAs have also been associated with 3′UTR of *SIRT1*, such as *miR-34a*, *miR-132*, and *miR-199a* and this association is tissue specific and results in downregualtion of *SIRT1* expression in colon, adipocyte, and cardiac tissues, respectively [39,43]. Another study using gastric cancer cell in a mouse model, showed overexpression of *miR-499* resulted in decreased expression of *SIRT1* which resulted in 60% growth inhibition when compared with control. This was further shown using FACS analysis and β-Gal activity assays. Importantly, qPCR analysis also showed a loss of *miR-499* expression in human clinical gastric tumor when compared with normal tissue [44]. Moreover, SIRT-1 protein level was found to be higher in mouse embryonic stem cells when compared with mouse differentiated tissues. Certain classes of miRNAs such as *miR-9*, *miR-181a*, *miR-181b*, *miR-204*, *miR-199b*, *miR-135*, post-transcriptionally downregulate *SIRT1* levels in differentiated tissues [45]. Further, in support of a tumor suppressor role of miRNAs, a study was performed on T24 bladder cancer cells in which cells were treated with a DNA demethylating agent and HDAC inhibitors, which resulted in a decrease in DNA methylation and an increase in histone activation around the promoter region of the *miR-127* gene. This ultimately lead to increased expression of *miR-127* and tumor inhibition [46].

## miRNA modification by diet

Growing evidence suggests that bioactive dietary components impact epigenetic processes and are often involved with the reactivation of tumor suppressor genes, activation of cell survival proteins, and induction of cellular apoptosis in many types of cancer [47-49]. Recent evidence suggests that bioactive dietary components can also target various oncogenic or tumor suppressive miRNAs to alter the gene expression profile in cancer prevention [50-52]. Genistein, an isoflavone isolated from soybeans, has been reported to have both preventive and therapeutic effects on carcinogenesis and many other diseases [50]. One of the studies performed on ovarian cancer cells, which compared treated and non-treated cells, found that there were a total of 53 miRNAs which were differentially expressed in the cancer cells. Further, upon analyzing gene expression data using real time PCR, both ER-α and ER-β were observed to be induced in genistein-treated cells, which can correlate with the expression changes of these 53 miRNAs, hence revealing a significant reduction in migration and invasion of ovarian cancer cells. Another investigation used dietary genistein for treatment of uveal melanoma cells and found a time-and dose-dependent inhibition which might be due to inhibition of *miR-27a *[51].

Curcumin (diferuloylmethane), a naturally occurring flavinoid derived from the rhizome of *Curcuma longa*, has been reported to alter the expression of miRNAs. One such study performed by Sun et. al. found an upregulation of *miRNA-22* and down-regulation of *miRNA-199a* in a pancreatic cancer cell line and a similar study was performed using curcumin in a breast cancer cell line which showed an upregulation of *miRNA-15a* and *miRNA-16* using real time PCR analysis [53].

Epigallocatechin-3-gallate (EGCG) is a major component of green tea and is thought to exert its anticancer effects by epigenetic mechanisms [54]. Studies have shown that EGCG can inhibit epigenetic enzyme activity and thus can modulate apoptosis, the cell cycle and cell proliferation. Recently EGCG is also found to modulate miRNA expression. To further confirm this notion, a study was performed using hepatocellular carcinoma cells and it was found that there is an increase in *miR-16* expression which resulted in apoptosis [55].

## Conclusion

miRNAs, a small group of noncoding RNAs, are drawing more attention than ever and are thought to be a new category of tumor suppressors or mediators of signal transduction. Further studies are needed to understand the interactions and regulatory mechanisms between miRNAs and their target molecules. Therefore, it will be important to clarify how the miRNA/*SIRT1*/*p53*/*DNMT* regulatory network is controlled in humans in future research (Table 2). Recent studies have suggested that miRNAs may act as tumor suppressors by regulating various cellular phenomenona like apoptosis, cellular movement, metastasis and cell proliferation. The regulatory loop between *SIRT1* and miRNA might provide new opportunities for therapeutic tissue-specific regulation and cancer inhibition. However, the mechanism by which miRNA regulation occurs is still unclear. Besides the involvement of miRNA in cancer, miRNAs may also influenced the aging process and provide a new avenue for potential targets in aging biology. miRNAs are also investigated as early plasma biomarkers and are expected to be more sensitive when compared with current biomarkers. From a clinical perspective, miRNAs can induce diverse effects but care must be exercised when extrapolating findings from *in vitro* to *in vivo*. Despite difficulties to overcome, the value of miRNAs in clinical applications is projected to be monumental.

**Table 2:** The effects of miRNA on target molecules

**Target molecule in cancer**	**miRNA involved**	**Effects on target molecule**
SIRT1/HDAC III	*miR-34a, miR-22, miR-499, miR-200, mi-9, miR-181a, miR-181b, miR-204, miR-199b, miR-135*	Down-regulated
p53	*miR-34a*	Upregulated
EMT	*miR-200*	Down-regulated
DNMT	*miR-148, miR-29a, miR-29b, miR-29c*	Overexpression of DNMTs
EZH2 histone lysine methyltransferase	*miR-101*	Upregulated and causes drug resistance in breast cancer
ER expression	*miR-101, miR-206, miR-221/222*	Down-regulated
p21	*miR-34a*	Upregulated

## Abbreviations

miRNA: Micro RNA; DNMT: DNA methyltransferases; HDAC: Histone deacetylases; HAT: Histone acetylases; SIRT: Silence information regulatory; UTR: Un-translated region; RISC: RNA-induced silencing complex; MESC: Mouse embryonic stem cells; SVR: Support vector regression; NAD: Nicotinamide adenine dinucleotide; FACS: Fluorescence activated cell sorter; EMT: Epithelial to mesenchymal transition; EGCG: Epigallocatechin-3-gallate; PCR: Polymerase chain reaction; 5-FU: 5-fluorouracil; ER: Estrogen receptor.

## Competing interests

No potential competing interests were disclosed.

## Authors’ contributions

Primary author: RK; Author of bioinformatics section: GWP; Edited the manuscript: TMH and TOT. All authors read and approved the final manuscript
